# Congenital Infantile Myofibroma: The Importance of Molecular Diagnosis

**DOI:** 10.7759/cureus.35885

**Published:** 2023-03-08

**Authors:** Joana Machado Morais, Carolina Castro, Cláudia Ferraz, Jorge Lima, Helena Barroca, Maria Bom-Sucesso

**Affiliations:** 1 Pediatrics, Hospital Pedro Hispano, Porto, PRT; 2 Pediatrics and Neonatology, Hospital Pedro Hispano, Porto, PRT; 3 Molecular Biology, Instituto de Patologia e Imunologia Molecular da Universidade do Porto, Porto, PRT; 4 Anatomic Pathology, Centro Hospitalar Universitário São João, Porto, PRT; 5 Pediatric Oncology, Centro Hospitalar Universitário São João, Porto, PRT

**Keywords:** treatment choices, next generation sequencing (ngs), core needle biopsy, molecular oncology, solitary infantile myofibroma

## Abstract

Infantile myofibromatosis is an uncommon soft tissue neoplasm that may present at birth or in early infancy. Although rare, this neoplasm is one of the most common benign fibrous tumors of infancy. Even though these tumors do not spread, they can compress or damage nearby organs. There is not an established management protocol, but it is advisable to maintain periodic clinical and imagological control until stability. Watchful waiting is an option to consider in the absence of problematic symptoms and visceral involvement. We report a case of solitary infantile myofibromatosis, without visceral involvement. It showed an initial rapid growth, raising concern among medical doctors and motivating soft tissue biopsy, always recommended as the clinical picture deviates from the classic presentation. Histology interpretation is often challenging, making genetics and clinical evaluation essential to exclude and prevent the misdiagnosing of more aggressive lesions.

## Introduction

Infantile myofibromatosis (IM) is an uncommon soft tissue neoplasm that may present at birth or in early infancy. Although rare, this neoplasm is one of the most common benign fibrous tumors of infancy [1-4]. Even though these tumors do not spread, they can compress or damage nearby organs. IM can present as a solitary lesion or multicentric involvement. Clinically solitary infantile myofibroma presents as a painless firm cutaneous or subcutaneous nodule. Most cases occur spontaneously, but familial forms associated with germline mutations of PDGFRB are described [5,6]. There is not an established management protocol, but it is advisable to maintain periodic clinical and imagological control until stability. Treatment options depend on the characteristics (size, number, and location) of the lesions as there are no standardized treatment protocols or guidelines. Watchful waiting is an option to consider in the absence of problematic symptoms and visceral involvement [1-4].

## Case presentation

**Figure 1:**
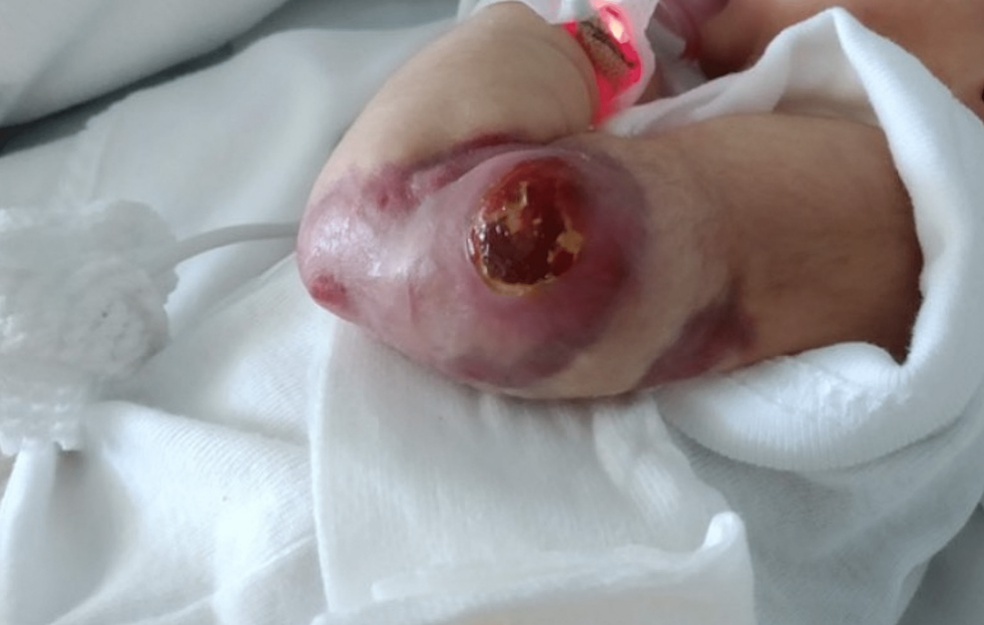
Clinical presentation Firm purple nodule of 3 cm in diameter, located in the skin and subcutaneous cellular tissue of the posterior-inferior 1/3 aspect of the left arm and elbow, with hemorrhage and ulceration.

A female newborn was delivered at 40 weeks to a 30-year-old gravida 1, para 0. Her family history was unremarkable. Pregnancy was uneventful and no fetal anomalies were detected at the prenatal ultrasound. The mother underwent a vacuum-assisted delivery. Apgar score was 9, 10, and 10 at 1, 5, and 10 minutes, respectively. Anthropometric measurement at birth was appropriate for gestational age. The postnatal exam showed a firm purple nodule on the skin and subcutaneous layer, with 3 cm of diameter, of the inferior 1/3 and posterior aspect of the left arm and elbow, with hemorrhage and ulceration (Figure 1).

**Figure 2:**
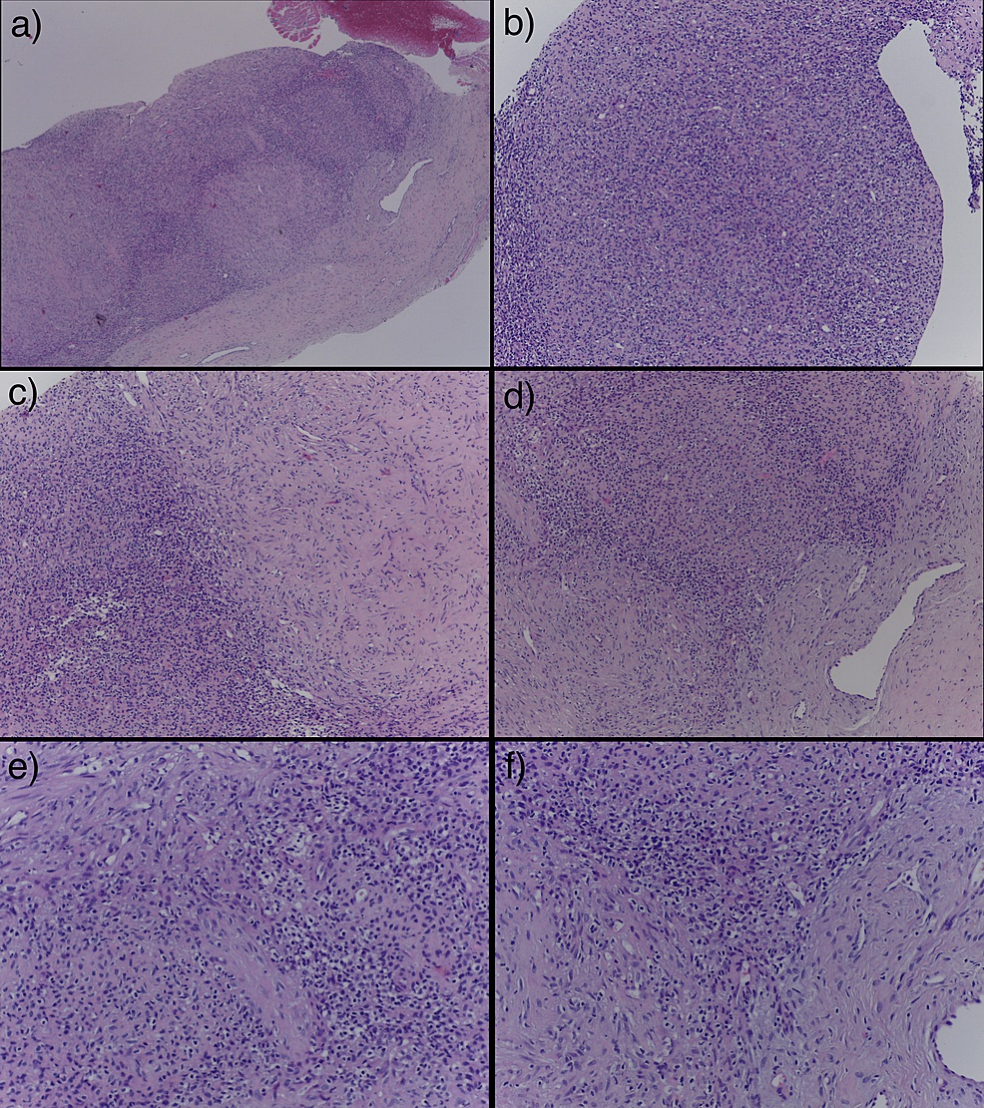
Histology Figures 2a (HE 40x) and 2b (HE 100x) - Core needle: on histology, a neoplasm with a solid pattern and hypocellular areas, collagen-rich alternating with others with higher cell density is seen; Figures 2c and 2d - (HE100x) - The neoplasm was constituted by an alternation of more fusiform hypocellular areas with more cellular and epithelioid areas; Figures 2e and 2f - (HE200x) - Focal pleomorphism is better remarked in higher magnification.

Ultrasound suggested an angiosarcoma. Magnetic resonance imaging (MRI) depicted a high-density mass (42x30x13mm) on T2 weighted images with a feeding vessel near the humerus, suggesting a vascular tumor, without a bone lesion. A core biopsy was performed, and histology displayed a spindle and epithelioid cell tumor with a solid pattern. It also showed hipocelular hypocellular areas, collagen-rich alternated with others with higher cell density, and more epithelioid phenotype (Figure 2). Neoplastic cells showed focal pleomorphism, but no necrosis or mitoses were seen (Figure 2).

**Figure 3:**
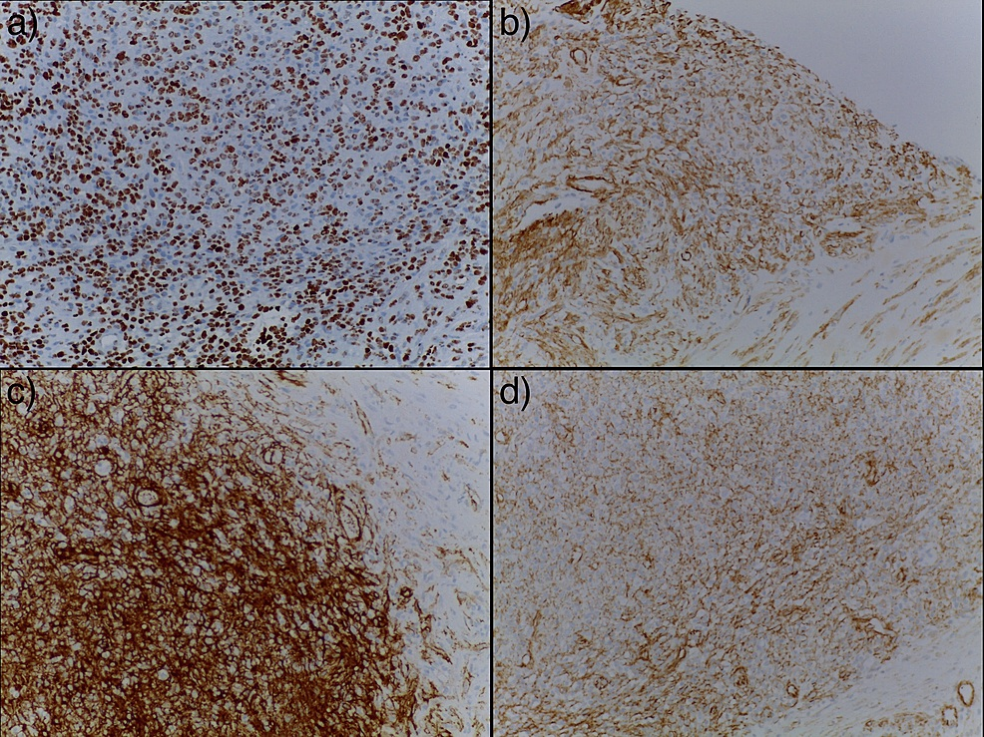
Immunohistochemical staining (200x) - Immunostains - (a) Actin; (b) Caldesmon; (c) CD34; (d) Beta Catenin - only cytoplasmic stain was seen

Focal calcification was seen. Immunoreactivity with smooth muscle actin (SM), caldesmon, and CD34 was strong and diffuse. No nuclear stain with beta-catenin was seen and no expression was detected with S100, EMA, STAT-6, desmin, CD30, or ALK (Figure 3).

**Figure 4:**
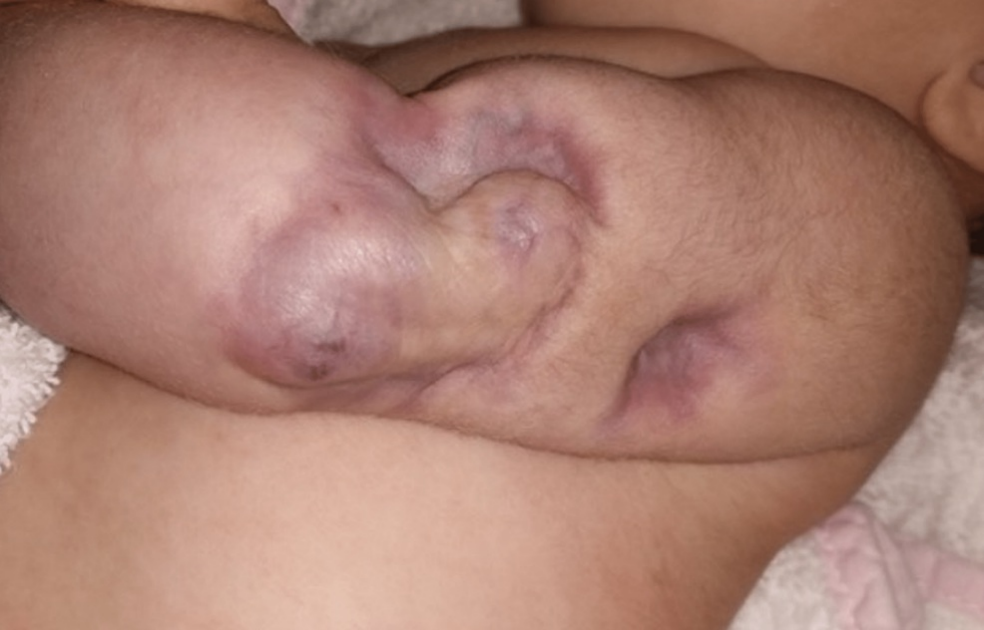
Lesion involution The regression after four months.

The diagnosis of an infantile myofibroma was suggested. A molecular study was performed using the NGS panel Oncomine^TM^ Childhood Cancer Research Assay to search for molecular alterations at the DNA (SNVs, indels, and CNVs) and RNA (gene rearrangements) levels. The panel includes comprehensive full exon mutation coverage for 140 genes, including PDGFRB, while also detecting rearrangements in 97 genes, including NTRK1, NTRK2, NTRK3, BRAF, RAF1, ALK, ROS1, among many others. The panel did not reveal any molecular alteration, including ETV6::NTRK3, lowering the probability of an infantile fibrosarcoma diagnosis. Thoraco-abdominopelvic computed tomography (CT) excluded asymptomatic lesions and cardiac and cranial ultrasounds were normal. After an initial rapid growth in the first 15 days of life, the lesion started to slowly involute. The lesion continued to regress without any treatment and, after four months, it completely involuted, without local atrophy but with no mobility impairment (Figure 4).

## Discussion

IM is a rare benign tumor characterized by single or multiple soft tissue nodular lesions [1,2]. A painless firm cutaneous or subcutaneous nodule is the characteristic presentation of solitary infantile myofibroma, as seen in the described case. However, the presence of bleeding and ulceration, not always present, motivated the exclusion of other differential diagnoses, such as infantile fibrosarcoma. Most cases occur spontaneously, but familial forms associated with germline mutations of PDGFRB are described, mainly in the multicentric form of the disease [5,6]. The patient did not have mutations of the PDGFRB gene in the NGS study. Once clinical presentation can be misleading, the diagnosis should have histologic confirmation [3]. Its interpretation is often challenging, making genetics and clinical evaluation essential to exclude and prevent the misdiagnosing of more aggressive lesions. The NGS panel did not reveal any molecular alteration, including ETV6::NTRK3, lowering the probability of an infantile fibrosarcoma diagnosis.

Solitary lesions, as the one described, have not been associated with a fatal outcome as they usually course with spontaneous resolution [3,4]. Still, as Mashiah et al. described, visceral involvement is associated with significant morbidity and high mortality. There are no established management protocols, but it is advisable to maintain periodic clinical and imagological control until stability and after, as recurrence has been reported. Surgical excision is the main treatment option, usually reserved for tumors that compress or damage nearby organs [4]. The treatment options depend on the lesion's characteristics (size, number, and location), as there are no standardized treatment guidelines. Zaoh et al. evaluated 32 cases of IM and concluded that watchful waiting can be contemplated for IM without visceral involvement or problematic symptoms [4]. We report a case of solitary IM, without visceral involvement. It showed an initial rapid growth, raising concern among medical doctors and motivating soft tissue biopsy, always recommended as the clinical picture deviates from the classic presentation. No treatment was done in this case, as it involuted spontaneously.

## Conclusions

IM should be part of the differential diagnosis of any solitary or multiple tumors in the soft tissues. The diagnosis must be confirmed histopathologically, as the clinical presentation can be misleading. Treatment options should be weighted case by case since there are no established management protocols or guidelines. Solitary lesions have a good prognosis, with spontaneous regression usually occurring in the first few years after diagnosis.
